# Acute Ischemic Stroke Treatment, Part 2: Treatment “Roles of Capillary Index Score, Revascularization and Time”

**DOI:** 10.3389/fneur.2015.00117

**Published:** 2015-06-01

**Authors:** Firas Al-Ali, John J. Elias, Danielle E. Filipkowski

**Affiliations:** ^1^Department of Neuro Interventional Surgery, Akron General Medical Center, Akron, OH, USA; ^2^Department of Research, Akron General Medical Center, Akron, OH, USA

**Keywords:** acute ischemic stroke, intra-arterial treatment, revascularization, stroke outcome, capillary index score

## Abstract

Due to recent results from clinical intra-arterial treatment for acute ischemic stroke (IAT-AIS) trials such as the interventional management of stroke III, IAT-AIS and the merit of revascularization have been contested. Even though intra-arterial treatment (IAT) has been shown to improve revascularization rates, a corresponding increase in good outcomes has only recently been noted. Even though a significant percentage of patients achieve good revascularization in a timely manner, results do not translate into good clinical outcomes (GCOs). Based on a review of the literature, the authors suspect limited GCOs following timely and successful revascularization are due to poor patient selection that led to futile and possibly even harmful revascularization. The capillary index score (CIS) is a simple angiography-based scale that can potentially be used to improve patient selection to prevent revascularization being performed on patients who are unlikely to benefit from treatment. The CIS characterizes presence of capillary blush related to collateral flow as a marker of residual viable tissue, with absence of blush indicating the tissue is no longer viable due to ischemia. By only selecting patients with a favorable CIS for IAT, the rate of GCOs should consistently approach 80–90%. Current methods of patient selection are primarily dependent on time from ischemia. Time from cerebral ischemia to irreversible tissue damage seems to vary from patient to patient; so focusing on viable tissue based on the CIS rather than relying on an artificial time window seems to be a more appropriate approach to patient selection.

## Introduction

The interventional management of stroke (IMS) III trial (1) showed non-superiority of intra-arterial (IA) revascularization combined with intra venous (IV) tissue plasminogen activator (tPA) treatment over IV tPA alone, and the systemic thrombolysis for acute ischemic stroke (SYNTHESIS) trial demonstrated similar lack of favorable clinical outcomes for IA versus IV tPA therapy (2). This is despite the high revascularization rate in the IA arms in these trials. The role of intra-arterial treatment for acute ischemic stroke (IAT-AIS) has been contested. Paradoxically, however, the benefit of revascularization to clinical outcomes is convincingly attested to in prior literature. In a recent meta-analysis of 998 patients with clinical follow-up at 3 months, good clinical outcome was found in 58% of revascularized patients as compared to 24.8% in non-revascularized patients (3). When revascularization occurred within the first 6 h, good clinical outcomes (GCOs) were found in 50.9% of revascularized patients as compared to 11.1% in non-revascularized patients. Other authors reached similar conclusions. Even in the IMS III trial, better revascularization using the modified thrombolysis in cerebral infarction (mTICI) score led to better outcomes than those for patients who achieved lesser revascularization (1). This data were recently resolved with the publication of newer trials. In MR CLEAN, EXTEND-IA, and ESCAPE, good recanalization rates were achieved in 58.7, 86, and 72.4% of patients, respectively, with accompanying GCO rates at 32.6, 71, and 53%, respectively (4–6). While these results demonstrate IA superiority with higher recanalization rates than with IVT, there are still a significant number of patients who achieved good and timely revascularization that did not also achieve GCOs. So if better revascularization improves outcome and IA treatment has a better revascularization rate than IV treatment, how can we explain the lack of GCOs in some of these patients?

## Revascularization and Outcome

Revascularization is defined as the restoration of anterograde blood flow to the ischemic area through the recently occluded artery. Currently, this is reported using the mTICI score, with mTICI of 2b or 3 being considered successful revascularization (7). The aim of revascularization is to produce clinical improvement through restoring the cerebral blood flow (CBF) level to greater than the critical threshold of 23 ml/100 g/min of viable brain tissue (8). This should translate into a permanent resolution of AIS symptoms by saving the ischemic tissue *before* it progresses to irreversible damage. So if perfect revascularization is achieved (mTICI = 3) in a *timely* manner, i.e., before ischemia becomes irreversible, clinical improvement should be achieved for almost all patients, as well as for the majority of patients with less effective revascularization (mTICI = 2b). However, review of the literature reveals that only around 50% of patients in whom we obtained timely recanalization (mTICI 2b, 3) will achieve a good clinical outcome (Table 1) (1, 2, 9–13). Attempting to solve the paradox regarding why all technically successful revascularizations do not translate into GCOs should help us improve our revascularization strategy.

**Table 1:** **Clinical outcomes across IAT-AIS trials**.

Trial	% mRS 0–2 (3 months)	Time to IAT (h)	% TIMI 2, 3
PROACT II	42.3^a^	4.5^b^	58
IMS I	43	3.05 ± 0.8^b^	56
IMS II	46	n/a	64
IMS III	40.8	3.5^b^	81^d^
SYNTHESIS	41.9	3:45^c^	n/a
SWIFT	37	4.9^b^	83
TREVO 2	39.9	4.7^c^	90
MR CLEAN	32.6	4.3^c^	58.7^d^
EXTEND-IA	71	3.5^c^	86^d^
ESCAPE	53	3.1^c^	72.4^d^

*^a^Barthel index 9 and 10*.

*^b^Mean*.

*^c^Median*.

*^d^TICI 2,3 for M1 occlusion*.

## Revascularization Rate

Revascularization rate depends heavily on the mode of treatment used (3). Spontaneous recanalization is estimated at 24% within the first 24 h (3). By comparison, overall data suggest that IV tPA results in recanalization in 46% of patients, as compared to 63% for IA thrombolysis, and 68% when the combined therapies (IV + IA) are utilized. Mechanical thrombectomy achieved the highest recanalization rate at 84% (3). It is estimated that revascularization is associated with a four to fivefold increase in good clinical outcome rates. Since higher revascularization rates correlate with better outcome in the literature and mechanical thrombectomy has the highest revascularization rate, it is now the preferred method for most operators.

## Mechanical Thrombectomy

The original method of mechanical thrombectomy was micro-wire and micro-catheter clot manipulation during IA tPA or pro-Urokinase infusion. In Asia, balloon angioplasty is used frequently as a mechanical method with an excellent recanalization rate of 80% (3). In the Western hemisphere, while balloon angioplasty is used, the predominate mode of mechanical recanalization is either a stent retrieval or the Penumbra system.

Stent retrieval systems are designed to restore blood flow by catching the thrombus through the stent struts. Flow cessation is then induced in the internal carotid artery using a balloon-mounted guiding catheter. At this time, the clot is removed by dragging it through the guiding catheter while applying suction on the guiding catheter to decrease the chance of a clot fragment migrating downstream. There are two available stent retrieval systems in the market today: the Trevo™ Pro Vu™(Stryker, Kalamazoo, MI, USA) and the Solitaire™(Covidian, CA, USA). Both devices are constructed of Nitinol with a laser cut design that can be delivered through a standard 0.021 or 0.027-inch (internal diameter) microcatheter.

The Trevo™ ProVue™ consists of a flexible, tapered core wire with a shaped section at the distal end. Radiopaque platinum wires in the shaped section and a guide wire-like tip allow fluoroscopic visualization. It is constructed of a straight cut tube that includes a distal taper and wire. Its struts are constructed perpendicularly to the clot in an attempt to engage the thrombus. The Solitaire™, on the other hand, has a proprietary overlapping stent technology called Parametric™ Design that provides multiple planes of clot contact (Solitaire IFU). Both stents have demonstrated comparable and excellent revascularization rates in prospective registry studies. In a recent prospective study of 227 patients, the Solitaire™ system had excellent results of 71% mTICI 2b or 3 (14), while the Trevo™ Pro Vu™ demonstrated 86% TICI 2 or 3 revascularization in the Trevo versus Merci retrievers for a thrombectomy revascularization (TREVO 2) randomized trial (13).

The Penumbra System™(Penumbra Inc., Fremont, CA, USA) is an aspiration system that utilizes an entirely different mechanism of mechanical clot retrieval. The device uses a suction mechanism to retrieve the clot inside the catheter by lodging the tip of the catheter in the proximal end of the clot while simultaneously hooking its hub to a suction machine creating pure suction (−29 mm Hg at sea level). In the initial pivotal study that included 125 patients, recanalization rates utilizing the Penumbra system were 82% thrombolysis in myocardial infarction (TIMI) score of 2 or 3 (15), later confirmed by a second prospective trial with 87% revascularization rates (TIMI 2 or 3) (16).

## From Technically Successful to Clinically Beneficial Revascularization

Technically successful revascularization does not always lead to good, i.e., beneficial, clinical outcomes. Some technically successful revascularizations are futile (not followed by clinical improvement) while others are outright harmful (cause clinical deterioration). Several factors may contribute to these variations:

### Patient selection: The capillary index score

In patients who already suffered a large area of irreversible ischemic injury, reconstituting the anterograde blood flow will not be beneficial, and can actually be harmful by increasing the risk of vasogenic edema and/or hemorrhagic transformation, as well as possible herniation. We believe one reason why good revascularization does not always lead to good clinical outcome is poor patient selection, i.e., treating patients with already irreversible ischemia. The capillary index score (CIS) is a simple angiography-based scale for assessing viable tissue in the ischemic territory. The CIS is comprised of a 4-point scale ranging from 0 (no angiographic capillary blush) to 3 (the whole ischemic area exhibits capillary blush), with the presence of capillary blush proposed as a marker of residual viable tissue, with absence implying irreversible ischemia. Favorable CIS (*f*CIS) is defined as a score of 2 or 3 and was found to be nearly a prerequisite for a good clinical outcome (modified Rankin Scale, mRS, score of 2 or lower at 90 days) (17), whereas a poor CIS (*p*CIS) is defined as a score of 0 or 1. If the assumption that the presence of *capillary blush indicates viable tissue* and its absence implies irreversible ischemia is correct, then selecting only patients with *f*CIS for treatment should significantly increase the percentage of patients with GCOs following technically successful intervention. At the same time, by not offering treatment to patients with *p*CIS, there should be a significant decrease in the percentage of futile or harmful revascularization, further increasing the percentage of patients with GCOs. Indeed, in the Borgess Medical Center-acute ischemic stroke registry (BMC-AIS), 83% of patients with *f*CIS who achieved TIMI 3 revascularization had good clinical outcome (mRS 0–2) (17). In a subgroup analysis of IMS I, II trials using the CIS and TIMI scores, 100% of the five patients with a *f*CIS and good revascularization (mTICI 2b, 3) had good clinical outcome (18). To our knowledge, this represents the highest percentage of GCOs following good revascularization that has been reported, suggesting that the CIS is the most accurate tool, to date, for patient selection in AIS treatment.

### Territory selection: Complete versus optimal revascularization

The current understanding of revascularization is that clinical benefits of revascularization increase with its extent (1, 19). In the IMS I and II trials, better revascularization led to better outcome – 46 versus 58% for TICI 2 or 3 versus mTICI 2b or 3, respectively (19). Even in the IMS III, despite its overall results, better revascularization in the IA arm translated into better clinical outcomes (Table 2) (1). However, if we accept the assumption that capillary blush indicates viable tissue, we should not be guided solely by the desire to obtain as complete revascularization as possible. Rather, the aim of revascularization should be to reconstitute anterograde flow *solely* to the territory with persistent capillary blush through the pial collaterals (viable tissue), while resisting the temptation to establish an anterograde flow to the territory void of capillary blush (non-viable tissue). In other words, and counter-intuitively, for a technically successful revascularization to be clinically beneficial, it does not necessarily need to be as complete as possible, but rather it should aim to restore an anterograde flow *only* to the area with persistent capillary blush. Following revascularization, *one should not see capillary blush that did not exist prior to intervention*.

**Table 2:** **IMS III results – clinical outcome and revascularization status (1)**.

mTICI	mRS 0–2 at 3 months (%)
0	12.7
1	27.6
2a	34.3
2b	47.9
3	71.4

### Complication rates

All forms of intervention, no matter how simple, carry the risk of complications. IAT-AIS is a very complex and technically demanding procedure, and at times it requires clinicians to cross occluded vessels blindly without any road mapping or prior knowledge of the patient’s anatomy. Furthermore, most of these patients are advanced in age and have difficult vessels to navigate. Complications related strictly to the revascularization attempts certainly exist; some of them are obviously device-specific. Unfortunately, information is lacking about the actual complication rate during IAT-AIS. Since these patients are already symptomatic prior to intervention, it is difficult to reliably determine how much an unsuccessful intervention contributed to overall patient symptoms or functional outcome impairment during their hospital stay. The only prospectively available data on complication during the revascularization procedure comes from the Penumbra™ aspiration system with a 13% total complication rate in the Pivotal study (3% deemed serious) and 6% in the post study (15, 16). Complication rates of 3% with the Solitaire™ system were reported in a review article involving 13 prior papers comprised of 262 patients (20). This included five subarachnoid hemorrhages, two self-detachments of stent, one entanglement of stent, and one in-stent thrombosis. Currently, no published data regarding Trevo complications are available, but the rates are likely similar to the other devices. We can thus conclude that mechanical intervention devices carry approximately 5% complication rate, which would ultimately negatively affect the overall odds ratio of better outcomes following IAT-AIS. Decreasing the complication rate is mandatory if we want to increase the percentage of treated patients with GCOs.

## The Different Forms of Revascularization

There are three forms of technically successful revascularizations: beneficial, futile, and harmful. We believe that beneficial revascularization, i.e., revascularization followed by clinical improvement, occurs when revascularization is completed only on the areas with persistent capillary blush via collaterals (prior to intervention). The role of revascularization here is simply to reverse the retrograde flow supplying the ischemic area to anterograde flow and by doing so raise the CBF above the critical threshold of ischemia. Therefore, technically *successful and beneficial, revascularization* can be defined as: *reversing the flow to an ischemic area with persistent capillary blush, from retrograde to antegrade without complications*.

The other forms of revascularization are futile (no clinical improvement) and harmful (followed by clinical deterioration). These occur when revascularization is performed on an area void of capillary blush prior to intervention, i.e., to non-viable cerebral tissue, or due to a complication during a revascularization attempt.

In order to enhance the benefit of intra-arterial treatment (IAT), we need first to redefine our revascularization strategy by minimizing the performance of futile and harmful revascularization. To achieve this goal, we propose the following strategy: select patients correctly with *f*CIS and obtain as complete and timely revascularization as safely possible, *solely* to the viable tissue, i.e., the areas with persistent capillary blush.

## Intra-Arterial Versus Intra-Venous Treatment

The recent results of the IMS III (1) and SYNTHESIS (2) trials are most likely due to poor patient selection and high percentages of futile or harmful revascularizations. By adapting the CIS for patient selection and a more nuanced strategy for revascularization, we should consistently approach the 80–90% clinical improvement rate in the treated subgroup, as we saw in the BMC-AIS registry and the subgroup analysis of IMS I, II. This percentage cannot be reached using IV treatment alone due to the lower revascularization rate associated with IV treatment and its inability to assess the collateral supply prior to treatment, which will invariably lead to a higher percentage of futile and harmful recanalization.

## Time to Revascularization and Outcome

### The relationship of time to revascularization and outcome: Is it linear?

#### Selection Bias

A linear relationship between time from ictus to revascularization and outcome is suggested from few previous trials (21–25). However, it is important to note that a selection bias exists in these trials since a significant portion of patients are excluded either due to the presence of imaging evidence of counter-indication for AIS treatment (signs of irreversible brain damage) or due to an artificial time window. Hence, even if the relationship between time from ictus to recanalization and outcome is perfectly linear in this subgroup of patients, we cannot deduce from it the overall relationship between time and outcome for all patients presenting with AIS.

#### Literature Review

The suggested linear relationship between time and outcome is not supported by empirical data when we reviewed the recent IAT-AIS trials. Reviewing the most recent large, prospective trials, the IMS III (1) and SYNTHESIS (2), as well as the two most recent device studies, solitaire with the intention for thrombectomy (SWIFT) and Trevo 2, reveal an almost identical clinical improvement rate despite significant differences in time from ictus to treatment across these studies (Table 1) (1, 3, 12, 13). The SWIFT and the Trevo 2 trials had similar results with a percentage of good clinical outcome (mRS ≤2) at 37 and 40%, respectively; the mean time from ictus to treatment was 4.9 h in the SWIFT study, and the median for Trevo 2 was 4.7 h (12, 13). Both trials included patients up to 8 h from ictus (12, 13). Meanwhile, in the IMS III trial, the IV treatment had to start within 3 h from ictus, while the IA treatment had to start within 5 h and finish by 7 h post ictus; yet, the study operators reported almost identical results with 40.8% mRS 0–2 at 3 months (1). In addition, the SYNTHESIS trial had a shorter time from ictus to treatment (median of 3:45 h; range 3:14–4:20) with a similar percentage of mRS 0–2 at 42% of mRS 0–2 (2). If the relationship between outcome and time from ictus to revascularization was linear, we would expect a higher percentage of mRS improvement in the SYNTHESIS trial than the IMS III trial, and a higher percentage in the IMS III trial than the SWIFT and Trevo 2 trials; yet, all reported an almost identical good clinical outcome rate. Furthermore, there are numerous series reporting almost identical percentages of GCOs (around 40%) on patients treated after the traditional 6-, even up to 8-h window (25, 26). It is difficult to reconcile these observations with a linear relationship between time from ictus to revascularization and outcome.

## The Collateral Supply and the Logarithmic Curve of Time to Outcome

**Figure 1:**
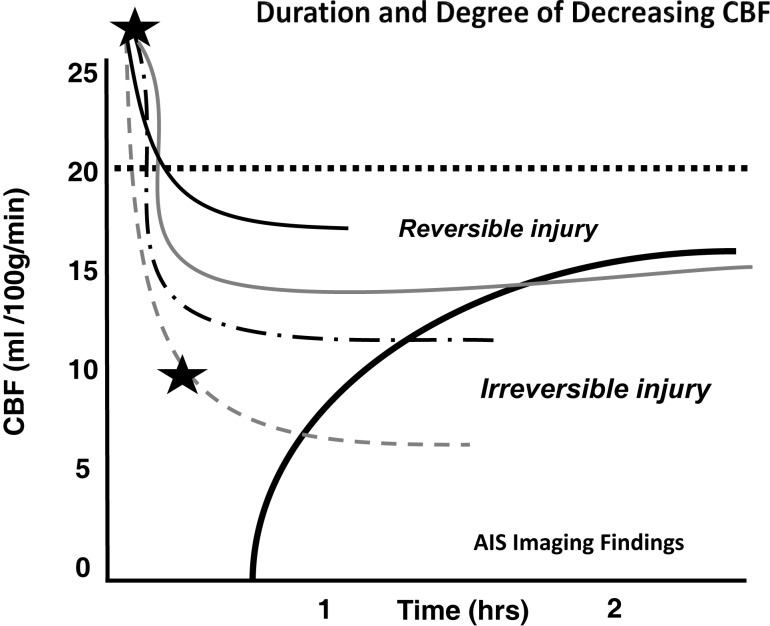
**Depth of ischemia and time to irreversible cerebral damage: time to irreversible cerebral damage depends on the depth of ischemia, which depends on the collateral supply**. Since different patients have different collaterals, the depth of ischemia will vary among patients, as will the time available for therapy to salvage the tissue (8). Adapted with permission from Jones et al., (8). Permission has been obtained from the American Association of Neurological Surgeons.

Crowell et al. have shown that following the arterial occlusion there is a sudden and abrupt drop in CBF (27). However, ischemia is never total and residual flow to the ischemic areas invariably persists through pial collaterals. Residual CBF (rCBF) will remain stable until revascularization occurs or cell death ensues. Studies have shown that time until ischemia becomes irreversible is heavily dependent on the rCBF, which varies depending on the collaterals present (8, 27). In other words, following cerebral ischemia, different patients will have varying amounts of time before cell injury becomes irreversible (Figure 1).

**Figure 2:**
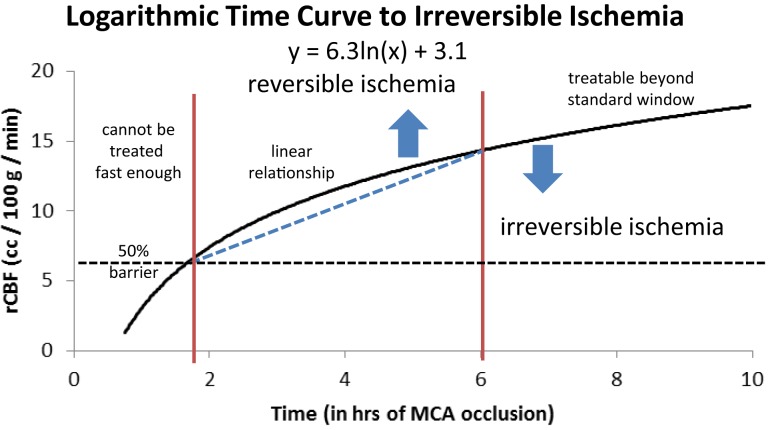
**Logarithmic time curve: the infarction threshold distinguishing between reversible and irreversible ischemia as a function of rCBF and time from ictus**. The vertical lines are an approximation and have not yet been validated (18). Reproduced with permission from Al-Ali et al. (18). Permission has been provided by Wolters Kluwer Health, Inc.

As we argued in a previous paper (18), when we consider a large cohort of patients with acute ischemic stroke we can grossly divide them into three groups depending on their rCBF. The first group will have such a low rCBF value that they will experience irreversible ischemia within an hour or two of ictus. For these patients, time to revascularization and its degree are irrelevant since the cerebral tissue will be irreversibly damaged by the time the patient arrives at the hospital. They are either not enrolled in studies due to evidence of ischemia on a computed tomography (CT) scan and other imaging modality, or do not improve following treatment despite timely and good revascularization (futile revascularization). We propose that approximately half of all AIS patients do not have sufficient collaterals to sustain ischemia until revascularization, no matter how fast it can be achieved, called “the 50% barrier.” A second group of patients will present with intermediate rCBF that will follow an approximately linear relationship between time to revascularization and outcome (a subtle gradual decrease). These patients are most often included in trials and registries. Finally, a third group has a higher rCBF than the others, but still below the critical symptomatic level of 23 ml/100 g/min (8). This group will exhibit a more asymptotic, flat curve relating time to revascularization and outcome, but they are usually excluded from studies when presenting outside the artificial time window. If we assemble these three groups as a whole, the relationship between time from ictus to revascularization and outcome will resemble a logarithmic function (Figure 2). In other words, if the patient has poor pial collaterals, no time will be fast enough. On the other hand, if pial collaterals are present and robust, we have longer time to revascularize the patient [not measured in minutes, but in hours (28)]. Simply put, if the patient has good collaterals they have time; if a patient has no collaterals they have no time.

## Patient Selection

The obvious implication of this logarithmic understanding of the time curve is the abandonment of any artificial time window to treatment since *each patient will have his or her own time* until irreversible ischemia occurs. Relying heavily on an arbitrary time window will significantly decrease the accuracy of patient selection in AIS treatment, either by including patients with irreversible ischemia just because they presented within the traditional time window and thereby leading to futile revascularization, or by denying treatment to patients who may still have viable tissue simply because they presented outside the traditional window. We propose a different patient selection algorithm, based more on objective signs of cerebral ischemia as opposed to an arbitrary time window.

## Proposed Patient Selection Algorithm

We recognize that the merit of the CIS still needs to be proven in a multicenter prospective study; however, we believe the CIS hypothesis will be proven true due to its ability to explain the results of the different ischemic stroke trials.

**Figure 3:**
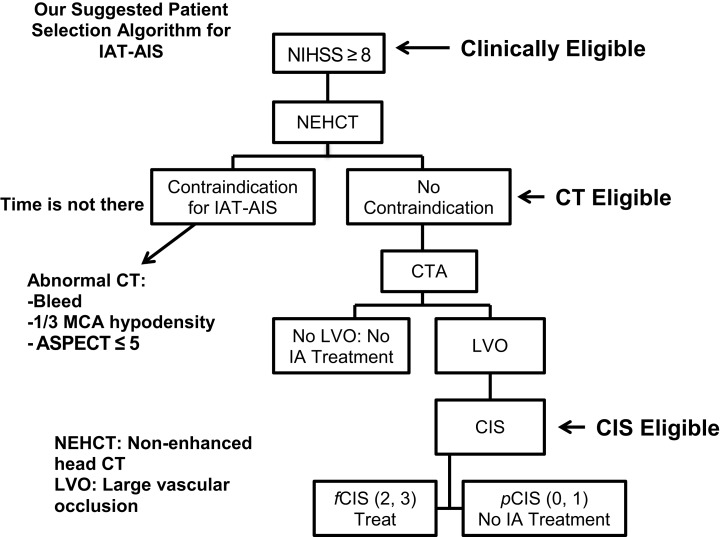
**Proposed patient selection algorithm for AIS**.

Since most patients will improve to a variable degree with time and physical therapy, we believe that IAT should be offered to patients suffering from a large stroke (NIHSS >8), with the only exception being aphasia (Figure 3). Of those patients who are *Clinically Eligible*, a non-enhanced head CT is obtained followed by CTA. These non-invasive tests can first rule out stroke mimics and identify patients with already visible signs of structural changes due to large irreversible ischemia (i.e., hypodensity in >1/3 MCA territory on head CT). If no such findings are identified, CTA will help confirm the vascular occlusion and its location. Patients with no counter-indication to treatment *and* proven large vessel occlusion are offered IAT, *CT Eligible*. For these patients, a full DCA is performed to obtain the CIS. Only patients who demonstrate *f*CIS should be offered IAT since revascularization on patients with *p*CIS will be futile and possibly harmful, *CIS Eligible*. If these steps are taken, we predict a significant increase in the percentage of treated patients with GCOs by virtue of significantly decreasing the percentage of futile and harmful revascularization. It is important to note that time from ictus to presentation is not included in this proposed algorithm. Since we believe that as long the patients advance successfully from clinical, to CT, to CIS eligibility, they are good candidate for intervention, regardless of time from ictus to presentation.

## Conclusion

Revascularization is the best hope for AIS patients. It should aim to reverse the flow to an ischemic area with persistent capillary blush from retrograde to anterograde without complications. Time from cerebral ischemia to irreversible damage varies from patient to patient and depends on their pial collaterals. In other words, the importance of time is secondary to the presence of collaterals. We believe that the relationship between time from ictus to revascularization and outcome is not linear, but logarithmic. Every patient has his/her own time before irreversible ischemia is reached, so it is critical to dispose of the artificial time window.

## Conflict of Interest Statement

The authors declare that the research was conducted in the absence of any commercial or financial relationships that could be construed as a potential conflict of interest.
